# *Mycobacterium tuberculosis* Specific CD8^+^ T Cells Rapidly Decline with Antituberculosis Treatment

**DOI:** 10.1371/journal.pone.0081564

**Published:** 2013-12-04

**Authors:** Melissa R. Nyendak, Byung Park, Megan D. Null, Joy Baseke, Gwendolyn Swarbrick, Harriet Mayanja-Kizza, Mary Nsereko, Denise F. Johnson, Phineas Gitta, Alphonse Okwera, Stefan Goldberg, Lorna Bozeman, John L. Johnson, W. Henry Boom, Deborah A. Lewinsohn, David M. Lewinsohn, for the Tuberculosis Research Unit and the Tuberculosis Trials Consortium

**Affiliations:** 1 Department of Medicine, Oregon Health and Science University, Portland, Oregon, United States of America; 2 Department of Public Health and Preventive Medicine, Oregon Health and Science University, Portland, Oregon, United States of America; 3 Department of Pediatrics, Oregon Health and Science University, Portland, Oregon, United States of America; 4 Uganda-Case Western Reserve University Research Collaboration, Kampala, Uganda; 5 Department of Medicine, Portland VA Medical Center, Portland, Oregon, United States of America; 6 Department of Medicine, College of Health Sciences, Makerere University, Kampala, Uganda; 7 Tuberculosis Research Unit, Case Western Reserve University, Cleveland, Ohio, United States of America; 8 Tuberculosis Trials Consortium, Centers for Disease Control and Prevention, National Center for HIV/AIDS, Viral Hepatitis and TB Prevention, Division of Tuberculosis Elimination, Atlanta, Georgia, United States of America; The Ohio State University, United States of America

## Abstract

**Rationale:**

Biomarkers associated with response to therapy in tuberculosis could have broad clinical utility. We postulated that the frequency of *Mycobacterium tuberculosis* (Mtb) specific CD8^+^ T cells, by virtue of detecting intracellular infection, could be a surrogate marker of response to therapy and would decrease during effective antituberculosis treatment.

Objectives: We sought to determine the relationship of Mtb specific CD4^+^ T cells and CD8^+^ T cells with duration of antituberculosis treatment.

**Materials and Methods:**

We performed a prospective cohort study, enrolling between June 2008 and August 2010, of HIV-uninfected Ugandan adults (n = 50) with acid-fast bacillus smear-positive, culture confirmed pulmonary TB at the onset of antituberculosis treatment and the Mtb specific CD4^+^ and CD8^+^ T cell responses to ESAT-6 and CFP-10 were measured by IFN-γ ELISPOT at enrollment, week 8 and 24.

**Results:**

There was a significant difference in the Mtb specific CD8^+^ T response, but not the CD4^+^ T cell response, over 24 weeks of antituberculosis treatment (*p*<0.0001), with an early difference observed at 8 weeks of therapy (*p* = 0.023). At 24 weeks, the estimated Mtb specific CD8^+^ T cell response decreased by 58%. In contrast, there was no significant difference in the Mtb specific CD4^+^ T cell during the treatment. The Mtb specific CD4^+^ T cell response, but not the CD8^+^ response, was negatively impacted by the body mass index.

**Conclusions:**

Our data provide evidence that the Mtb specific CD8^+^ T cell response declines with antituberculosis treatment and could be a surrogate marker of response to therapy. Additional research is needed to determine if the Mtb specific CD8^+^ T cell response can detect early treatment failure, relapse, or to predict disease progression.

## Introduction

Modern tools such as the GeneXpert® (Cepheid; Sunnyvale, California, USA) and line probe assays allow for the rapid identification of *Mycobacterium tuberculosis* (Mtb) as well as genetic mutations associated with drug resistance in clinical specimens. However, precise tools to ascertain among those infected, who will progress to tuberculosis (TB) disease, or who, once disease has developed, will fail treatment are lacking. These tools would be useful both for individual patient care and for clinical trials. In both cases, biomarkers that reflect bacterial burden or response to therapy could serve in this role.

Host factors such as cytokines, chemokines, immune cells, antibodies to Mtb, and differential gene expression profiles have all been investigated as potential biomarkers [1], [2], [3]. It has been postulated that the frequency and phenotype of pathogen-specific T cells could serve as a dynamic biomarker early in treatment [4], [5]. Early studies, using an assay similar to the T-SPOT®.*TB* (Oxford Immunotec, Inc, Oxfordshire, UK) enzyme-linked immunospot assay (ELISPOT), linked the frequency of Mtb specific T cell responses with antigenic load [6]. However, commercial interferon gamma (IFN-γ) release assays (IGRAs: T-SPOT®.*TB* and QuantiFERON®; Qiagen Inc., Valencia, California, USA), cannot discern TB from latent TB infection (LTBI) [7], [8], two infection phenotypes that differ significantly in bacterial burden. Thus, it is not surprising that studies examining the role of IGRAs as a marker of TB treatment have yielded results with a wide dynamic range [9], [10], [11], [12], [13] _ENREF_8, making the clinical utility of IGRAs as a biomarker of response to therapy less clear.

We postulate that the poor correlation of IGRAs with treatment reflects the biological inability of the CD4^+^ T cell to discern differences in intracellular bacterial burden. IGRAs measure IFN-γ released by peripheral blood mononuclear cells (PBMC), which are dominated by CD4^+^ T cells [14]. In this regard, CD4^+^ T cells recognize antigen presented in the context of “professional”, MHC-II expressing antigen presenting cells, which may have sampled their antigen from either the intracellular or extracellular milieu. Conversely, CD8^+^ T cells necessarily recognize antigen derived from an intracellular environment and could serve as sensors of bacterial burden. In this regard, human CD8^+^ T cells preferentially recognize cells heavily infected with Mtb [15] and the magnitude of the CD8 response in animal models is correlated with bacterial load [16], [17], [18]. Further, young children with TB have a robust Mtb specific CD8^+^ T cell response, which is absent from the healthy matched cohort of children with extensive household exposure [19]. Taken together, we postulated that the number of Mtb specific CD8^+^ T cells, by virtue of their ability to respond to intracellular mycobacterial antigens, could be used as a surrogate marker of response to therapy and thus would decrease during effective antituberculosis treatment.

To study this question, we enrolled 50 HIV-negative subjects with AFB smear-positive pulmonary TB and measured the Mtb specific CD4^+^ and CD8^+^ T cell responses at three time points during antituberculosis treatment. Our data provide evidence that the number of Mtb specific CD8^+^ T cells, potentially by detecting intracellular mycobacterial antigen, and hence intracellular infection, declines with antituberculosis treatment and may be a surrogate marker of response to therapy. As a secondary analysis, to explore the differences in the Mtb specific CD4^+^ and CD8^+^ responses on antituberculous treatment, we sought to determine if baseline nutritional differences affected or altered the association between the Mtb specific CD8+ T cell response and treatment duration.

Some of these results have been previously reported in an oral/poster presentation [20].

## Materials and Methods

### Ethics Statement and Study Oversight

The study protocol was approved by the institutional review boards at the Joint Clinical Research Centre, Kampala, Uganda, University Hospitals Case Medical Center, Cleveland, Ohio, USA and U.S. Centers for Disease Control (CDC) and the Ugandan National Council for Science and Technology. Written informed consent was obtained from all study participants.

### Study Design

A prospective cohort study to evaluate microbial and immunologic surrogate markers in response to TB treatment was undertaken jointly by the CDC TB Trials Consortium (TBTC) and the NIAID's Tuberculosis Research Unit (TBRU) (TBTC Study NAA2m/DMID 08-0023) at the Uganda-Case Western Reserve University Collaboration sites in Kampala, Uganda. The study describing the microbial surrogate markers will be reported separately. Our study objectives were to evaluate the Mtb specific CD4^+^ and CD8^+^ T cell response during antituberculosis treatment.

### Participants and Procedures

Patients aged 18 to 60 years with suspected pulmonary TB presenting to the National TB Treatment Center at Mulago Hospital in Kampala, Uganda between June 2008 and August 2010 were evaluated. HIV-negative, sputum AFB smear-positive patients with cavitary disease, residing within a radius of 20 kilometers from the treatment center, no history of previous TB treatment, and a Karnofsky Performance Scale Score >50% (requires occasional assistance, but is able to care for most personal needs) [21], were invited to participate after providing informed consent. In choosing sputum smear positive patients with cavitary disease, initial TB biomarker studies usually focus on patients with high sputum bacillary load. The rationale for this cohort selection is as follows: (a) it increases the chance of detecting a difference in the biomarker during treatment, and (b) sputum smear positive patients with pulmonary TB are the most important patient population for TB treatment trials, where TB biomarkers would be of use. As to the representativeness of patients with cavitary TB of all patients with TB, the proportion of patients with cavitary disease at the time of diagnosis ranges from 40 to 87% [22], [23]. Additionally, TB treatment trials have indeed shown that treatments that work in smear positive patients with cavitary disease generally work in patients with less severe forms of pulmonary TB and most forms of extrapulmonary TB. At enrollment, a medical history and physical examination were performed including weight and height for calculation of BMI [24]. Sputa for AFB smear grade [25], culture on solid and liquid MGIT media, quantitative and semi quantitative culture on solid Middlebrook media were collected at baseline (week 0) and every 2 weeks during the intensive phase of treatment (baseline, 2, 4, 6, and 8 weeks) and monthly during the continuation phase (3–9 months). A positive AFB smear was graded from 1 to 4+. Posteroanterior chest radiographs (CXR) were performed at baseline and read by chest physicians at Mulago Hospital using a standardized scoring system considering the presence and size of cavitary lesions and the involvement of multiple lung fields to grade the radiographic severity and extent of disease. For extent of disease, predefined cut-offs of limited, moderate and extensive were used to characterize the CXR with moderate reflecting ¼ and extensive ½ of thoracic area involvement. Blood was drawn for complete blood count, serum aspartate aminotransferase, total bilirubin, and creatinine. In addition, blood was collected at baseline and weeks 8 and 24 for ELISPOT analysis. All patients received an American Thoracic Society (ATS)/Infectious Diseases Society of America (IDSA)/CDC-recommended anti-TB chemotherapy regimen consisting of two months of daily isoniazid, rifampicin, ethambutol and pyrazinamide followed by four months (or seven months if the patient's sputum cultures were positive after the first two months) of daily isoniazid and rifampicin with dosages adjusted for body weight [26]. All doses were supervised and follow up occurred every two weeks during the intensive phase and monthly during the continuation phase. Anti-TB drugs were obtained from VersaPharm, Inc., Marietta, GA, USA (rifampin) and Svizera Europe BV, Almere, the Netherlands (isoniazid, ethambutol and pyrazinamide), and manufactured under Good Manufacturing Practice. Patients were followed through the end of TB treatment.

### Media and reagents

Culture medium consisted of RPMI 1640 supplemented with 10% human sera, 5×10^−5^ M 2 ME (Sigma-Aldrich,http://www.sigmaaldrich.com/), and 2 mM glutamine (GIBCO BRL, http://www.invitrogen.com/). Peptides were synthesized by Genemed Synthesis (http://www.genemedsyn.com/). A single synthetic peptide pool consisting of 15-mers overlapping by 11 amino acids, representing Mtb specific proteins, CFP-10 and ESAT-6, was synthesized. Peptides were resuspended in DMSO, and 43 peptides were combined into one pool such that each peptide in the pool was at a concentration of 1 mg/ml. Peptide pools were stored at 4°C.

### IFN-γ Enzyme Linked Immunospot (ELISPOT) assay

To measure Mtb specific CD4^+^ and CD8^+^ T cell responses, overnight IFN-γ ELISPOT assays were performed as described previously [19], [27] using a single synthetic pool consisting of 15-mers overlapping by 11amino acids, representing Mtb specific proteins, CFP-10 and ESAT-6. To achieve CD4 and CD8 separation and ensure quality control on the separated fractions, CD8 and CD4/CD56 depleted cells were isolated using the Miltenyi magnetic bead general protocol as previously described [19]. Negative and positive controls consisted of cells cultured with medium alone or phytohemagglutanin (PHA, 10 µg/ml; EMD Biosciences, http://www.emdbiosciences.com/), respectively for quality assessment. All CD4 and CD8 T cell determinations were performed in duplicate and media (control) wells without antigen were performed in triplicate. IFN-γ producing Mtb specific T cells were enumerated per 250,000 T cells. All assays were performed at the Joint Clinical Research Centre in Kampala, Uganda.

### Statistical Analysis

The ex - vivo frequency of Mtb specific T cells was calculated per time point, per person, as the average number of spot forming units (SFU) for wells containing antigen and compared with the average SFU in the control wells. To account for well-to-well variance among technical replicates, a standard deviation of the media control was calculated. A positive ELISPOT assay was defined as one in which the Mtb specific T cell response was at least two standard deviations above the background control. If these criteria were met, the mean of the background was subtracted out to enumerate the Mtb specific T cell response per 250,000 T cells. A positive PHA response was defined as ≥100 SFU per well.

To assess the overall change in the Mtb specific CD4^+^ and CD8^+^ T cell response over 24 weeks across 3 time points during antituberculosis treatment, a negative binomial mixed model for over-dispersed spot forming counts was used, with significance at 0.05. A negative binomial model was chosen and is appropriate for (1) ELISPOT count data (as opposed to continuous data), and (2) correlations within subjects when using repeated measures study design. Goodness of fit tests were performed to check model specifications and potential overdispersion or underdispersion in the Poisson regression. The generalized estimating equation (GEE) approach was adopted to estimate standard errors for the correlated data and the quasi-likelihood under the independent model criteria (QIC) for GEE was used to define optimal covariance structure. [28]. The significance level (*p* value) for pairwise comparisons between time points (0–8 weeks, 0–24 weeks) were reported after adjustment for multiple comparisons using the Bonferroni method. The percent decrease over 24 weeks and from baseline to 8 weeks was calculated from L'Beta estimate for pairwise comparisons using the following formula (1 – e^β^). The overall change in the T cell response to PHA was performed using the negative binomial mixed model as described above. A univariate negative binomial model was used to assess the effect of BMI (categorical variable ≤17 as reference), gender (female as reference), age (continuous variable), AFB smear grade (0–4), and quantitative mycobacterial cultures (colony forming units (CFU)) on the Mtb specific T cell response at the baseline time point., If a *p* value was ≤0.1, the variable was included in the multivariate model with the principal variable of interest, treatment duration. To analyze the change in microbiologic burden among subjects at baseline and week 4, as measured by the log of colony forming units, the nonparametric Wilcoxon signed rank test was used. Statistical analysis was performed in SAS version 9.2 (SAS Institute Inc., Cary, North Carolina, USA) and graphing was performed in Statistica (StatSoft, Tulsa, Oklahoma, USA).

## Results

### Characterization of TB patient cohort

Fifty-one, HIV-negative subjects with pulmonary TB were invited to participate in the study and baseline characteristics were collected from 50 subjects. The mean age of the clinical cohort was 26.6±6.0 years (Table 1). Twenty-four percent (12/50) were female. The average BMI was 18.8±3.1. Fifteen (30%) were malnourished as defined by a BMI≤17 [24]. All subjects had AFB smear-positive, culture confirmed pulmonary TB. Eight subjects had pulmonary and extrapulmonary TB, including 5 with pleural involvement. For the sites of extra-pulmonary disease, 5 had pleural disease, 1 had mediastinal involvement, and 2 had disseminated disease. Over one-third reported fevers, night sweats and loss of appetite. Nausea, dizziness, joint pain, headache, vomiting, diarrhea, and numbness were less commonly reported (2–8%; data not shown). Ninety-eight percent of enrolled subjects had cavitation on initial CXR. The one individual without cavitation did not have blood drawn beyond baseline. Extensive radiographic involvement, greater than ½ of the thoracic cavity, was noted in 31 (62%) subjects. Over one-half of the subjects had a sputum smear grade of 4^+^ at entry. Two subjects were later determined to have drug resistant TB on initial susceptibility testing; one subject was resistant to isoniazid and rifampin and other was resistant to isoniazid only. Treatment of these two patients was modified according to National Tuberculosis Program guidelines. The data were analyzed with and without inclusion of these two subjects, and there was no difference in the results. Thus, these subjects were retained in the final analysis. One subject with drug susceptible pulmonary TB died of unknown causes. All subjects improved during the treatment interval by standard microbiologic measures (Table 2).

**Table 1:** Baseline Characteristics.

Characteristics			
Clinical			
		Females, n (%)	12 (24)
		Mean age (years ± standard deviation)	26.6±6.0
		Body mass index at baseline (kg/m^2^ ± standard deviation)	18.8±3.1
		Fever n (%)	18 (36)
		Night sweats n (%)	24 (48)
		Anorexia n (%)	19 (38)
		Extra-pulmonary TB n (%)	8 (16)
Radiographic n (%)			
	Cavitation		
		Absent	1 (2)
		Single or multiple, diameter <4 cm in aggregate	18 (36)
		Single or multiple, diameter ≥4 cm in aggregate	31 (62)
	Extent		
		Limited, lesions <1/4 of thoracic cavity	0 (0)
		Moderate, lesions <½ of thoracic cavity	19 (38)
		Extensive, lesions involving >½ of thoracic cavity	31 (62)
	Pleural disease		5 (10)
Total n (%)			50 (100)

**Table 2:** Microbiologic burden of *mycobacterium tuberculosis* in relation to duration of treatment.

Diagnostic Test	Time Point		
AFB smear grade n (%)	Baseline – Week 0	Week 8	Week 24
4+	29 (58)	0 (0)	0 (0)
3+	14 (28)	6 (12.2)	0 (0)
2+	5 (10)	7 (14.3)	0 (0)
1+	2 (4)	8 (16.3)	3 (7)
Negative	0 (0)	28 (57.2)	40 (93)
Missing data n	0	1	7
Mycobacterial growth on 7H10 solid media n (%)			
>200 col.	50 (100)	0 (0)	0 (0)
20–100 col.	0 (0)	3 (6.1)	0 (0)
<20 col.	0 (0)	10 (20.4)	0 (0)
No growth	0 (0)	36 (73.5)	40 (100)
Contamination	0	0	5
Missing data n	0	1	5
Total n (%) *	50 (100)	49 (100)	40 (100)

Definition of abbreviations: col.  =  colonies

* Total n (%) calculated less missing data and contamination

### Immune response to Phytohemeagglutinin (PHA) improves during treatment

Blood was drawn at baseline, week 8 and week 24 of treatment for 49, 48, and 42 subjects respectively and interpretable ELISPOT results were available from all subjects. Ten subjects did not have blood drawn for at least one of the three time points for ELISPOT analysis, and 3 of the 10 subjects were missing blood from the week 8 and week 24 time points. The median mitogen or PHA responses (SFU) for the CD4 and CD8 ELISPOT were 204 (interquartile range of 144–373) and 193 (interquartile range 91 – 336) respectively at baseline. Using the same approach to assess the Mtb specific T cell responses during treatment as described in the methods, the response to PHA increased significantly with duration of treatment for both the CD4^+^ and CD8^+^ T cells. For the CD4^+^ T cells, the average response (SFU) to PHA at baseline, week 8 and week 24 was 248, 298, and 323 respectively with a significant difference during treatment (*p* = 0.038). For the CD8^+^ T cells, the average response (SFU) to PHA at baseline, week 8 and week 24 was 211, 259, and 283 respectively, also demonstrating a significant difference during treatment (*p* = 0.005). All but 3 individuals had a detectable Mtb specific T cell response, greater than two standard deviations above the media at baseline.

### Mtb specific CD8^+^ T Cells Decline with Antituberculosis Treatment

**Figure 1:**
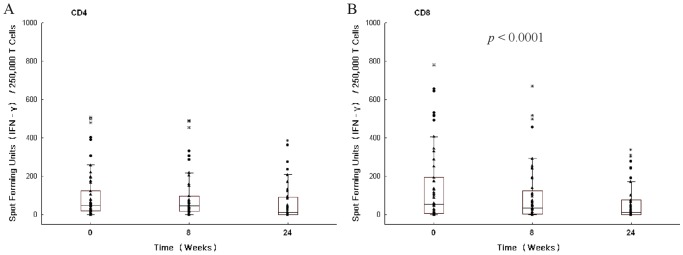
Magnitude of *Mycobacterium tuberculosis* (Mtb) specific T cell responses in smear and culture positive, HIV negative, pulmonary TB patients over time during antituberculosis therapy. The magnitude of the Mtb specific CD4^+^ T cell response (A) and Mtb specific CD8^+^ T cell response (B) is shown by IFN-γ ELISPOT in response to ESAT-6 and CFP-10 and reported in spot forming units per 250,000 T cells. Evaluable samples (n) analyzed per time point were 49, 48 and 42 for baseline, week 8 and week 24 respectively. A negative binomial mixed model was used to assess the overall change during 24 weeks. Raw data, surrounded by the box demonstrates the distribution of 25–75% of the data, with the whiskers representing the non-outlier data, and the horizontal line depicting the median. Raw data is shown with a triangle, outliers with a circle, and extremes with a star.

**Figure 2:**
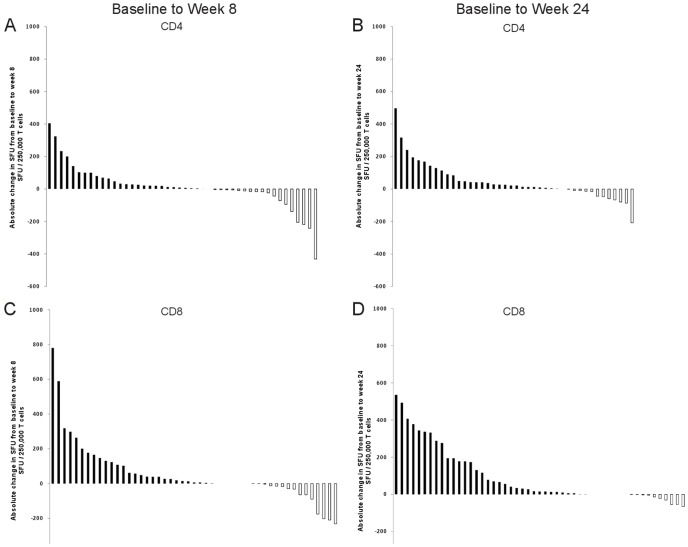
Individual profiles of the absolute change in the Mtb specific T cell responses (spot forming units; SFU) from baseline to week 8 and baseline to week 24 in smear and culture positive, HIV negative, pulmonary TB patients during antituberculosis therapy. The waterfall plots show the absolute change in magnitude (SFU) between the baseline time point to week 8 for the Mtb specific CD4^+^ (A) and CD8^+^ (C) T cell response. The absolute change in magnitude for the baseline time point to week 24 is shown for the Mtb specific CD4^+^ (B) and CD8^+^ (D) T cell response. Black bars represent SFU decreasing from baseline to the respective time point, whereas white bars represent increasing SFU from baseline to the respective time point.

We hypothesized that the frequency of Mtb specific CD8^+^ T cells could be used as a surrogate marker of response to therapy and would decrease during effective antituberculosis treatment. To test this hypothesis, we analyzed the Mtb specific CD4^+^ and CD8^+^ T cell response by IFN-γ ELISPOT in response to ESAT-6 and CFP-10 during antituberculosis treatment. Using a negative binomial mixed model, we first evaluated the overall change in the Mtb specific CD4^+^ and CD8^+^ T cell response over 24 weeks of antituberculosis treatment, independent of other variables. Taking into account all available time points (n = 3), there was a significant difference in the Mtb specific CD8^+^ T cell response during the 24 weeks of treatment (*p*<0.0001; Figure 1). Conversely, the Mtb specific CD4^+^ T cell response did not demonstrate a significant difference over 24 weeks of antituberculosis treatment. Individual profiles of the absolute change in the Mtb specific T cell responses from baseline to week 8 and baseline to week 24 are shown in Figure 2 and show the magnitude and direction of the changes in T cell responses between the time points.

### The effect of malnutrition on the Mtb specific T cell response during antituberculosis treatment

**Figure 3:**
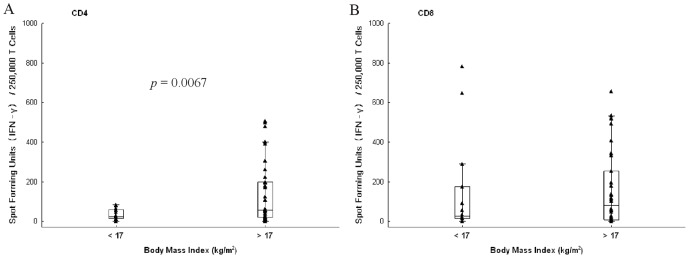
*Mycobacterium tuberculosis* (Mtb) specific T cell responses at baseline and BMI. The magnitude of the Mtb specific T cell response by IFN-γ ELISPOT to ESAT-6 and CFP-10, and reported in spot forming units/250,000 T cells, is shown at baseline for the Mtb specific CD4^+^ response (A) and the Mtb specific CD8^+^ response (B) for BMI≤17 (n = 14) and >17 (n = 34). A negative binomial model was used to assess differences in the Mtb specific T cell responses and BMI≤17 and >17. Raw data, shown as a triangle, surrounded by the box demonstrates the distribution of 25–75% of the data, with the whiskers representing the non-outlier data, and the horizontal line depicting the median.

**Figure 4:**
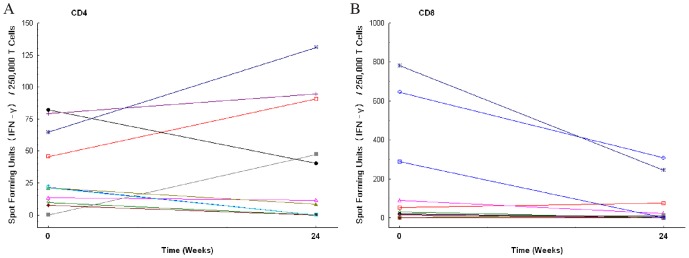
*Mycobacterium tuberculosis* (Mtb) specific T cell responses during antituberculosis treatment in subjects with baseline malnutrition (BMI≤17). The subgroup of subjects, who started therapy with a BMI≤17 with baseline and week 24 analyzable ELISPOT data (n = 11) are shown. The magnitude of the Mtb specific T cell response is shown by IFN-γ ELISPOT to ESAT-6 and CFP-10 and reported in spot forming units per 250,000 T cells. Connected lines at baseline and week 24 reflect a individual subject's profile for the Mtb specific CD4^+^ T cell response (A) and the Mtb specific CD8^+^ T cell response (B).

To explain this difference between the Mtb specific CD4^+^ and CD8^+^ T cell responses over the 24 weeks of treatment, we postulated that nutritional status could be differentially affecting Mtb specific CD4^+^ and CD8^+^ T cell responses. As a secondary analysis, we sought to determine whether an association between the Mtb specific CD4^+^ or CD8^+^ T response, if associated with time on treatment, might be correlated with malnutrition. In this regard, protein calorie malnutrition has been associated with reduced lymphocyte replication as well as a lower IFN-γ response in animal models [29], [30]. Furthermore, in a prior study looking at a HIV-negative cohort undergoing antituberculosis treatment, depressed IFN-γ in response to purified protein derivative was observed over a 12 month observation period [31]. To explore if the Mtb specific T cell response was affected by malnutrition, we used the BMI as a biomarker. Forty-eight subjects had both ELISPOT and BMI data for analysis at baseline. The Mtb specific CD4^+^ T cell response at baseline was significantly reduced in subjects with moderate to severe malnutrition (BMI≤17; n = 14) compared with subjects with a BMI>17 (n = 34; *p* = 0.0067). The Mtb specific CD8^+^ T cell response was less affected by baseline BMI (Table 3 and Figure 3). We postulated that during treatment, as BMI improved, the Mtb specific T cell response would improve. Eleven of the original 14 subjects with a baseline BMI≤17 had longitudinal data (Figure 4). In the subgroup of subjects that began antituberculosis treatment with a low BMI, no clear pattern emerges for the Mtb specific CD4^+^ T cell response, however, the Mtb specific CD8^+^ T cell response appears to decline for several subjects, as seen in the full cohort (Figure 4). We evaluated other covariates to assess the impact on the Mtb specific T cell response, including sex, age, baseline AFB smear grade, cavitary disease, and radiographic extent of disease. These covariates did not alter the Mtb specific T cell response thus were not included in the multivariate analysis.

**Table 3:** Univariate and multivariate analyses of predictors of the Mtb specific T cell response among HIV negative, smear positive, culture confirmed pulmonary TB patients undergoing antituberculosis treatment.

	CD4^+^ T Cell Response (*p value*)		CD8^+^ T Cell Response (*p value*)	
Predictor Variable	Univariate	Multivariate ‡	Univariate	Multivariate ‡
Treatment duration (weeks)	0.0582 ‡	0.0699	<0.0001 ‡	<0.0001
BMI * (ref≤17)	0.0067 †	0.0136	0.9286 †	0.4995
Gender (ref: female)	0.4271 †	n/a	0.8982 †	n/a
Age	0.1204 †	n/a	0.3667 †	n/a
AFB smear grade§ (ref: smear neg)	0.2975 †	n/a	0.2378 †	n/a
Extent || (ref: limited to moderate)	0.9007 †	n/a	0.3539 †	n/a
Cavitation ** (ref: ≤4 cm)	0.8037 †	n/a	0.7226 †	n/a

* BMI is included in the multivariate model with treatment duration.

† The association between variables and the Mtb specific T cell response was analyzed at the baseline time point.

‡ Statistical method: a negative binomial mixed model.

§ AFB smear grade 0 (smear negative) to 4+.

|| Extent: limited or moderate versus extensive by chest radiograph.

** Cavitation: ≤ 4 cm versus >4 cm.

n/a. not applicable.

To explore the association of malnutrition with the Mtb specific T cell response and treatment duration, we developed a multivariate model. Using a negative binomial mixed model, we evaluated the overall change in the Mtb specific CD4^+^ and CD8^+^ T cell response over 24 weeks of antituberculosis treatment, using BMI as a biomarker for nutritional status. Taking into account all available time points (n = 3), and BMI, the Mtb specific CD4^+^ T cell response was not significantly associated with treatment duration in the multivariate analysis (Table 3). In a parallel multivariate analysis, we analyzed the Mtb specific CD8^+^ T cell response adjusted for BMI. There was a significant difference in the Mtb specific CD8^+^ T cell response during the 24 week treatment interval (*p*<0.0001; Table 3). Furthermore, in a pairwise analysis, there was a significant difference from baseline to 8 weeks (*p* = 0.023) and from baseline to 24 weeks (*p* = 0.0003). The estimated Mtb specific CD8^+^ T cell response decreased by 58.4% at 24 weeks, with the majority of the decrease (38.7%) observed at 8 weeks.

### Neither smear grade nor quantitative cultures were associated with Mtb specific T cell responses

**Figure 5:**
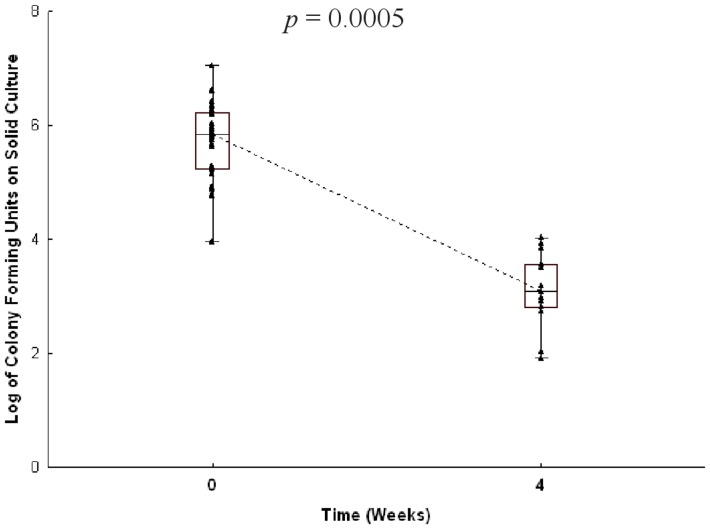
Quantitative cultures and treatment duration. Quantitative cultures from subjects with smear positive pulmonary TB as shown by the log of colony forming units (CFU) enumerated on 7H10 media at week 0 (n = 35) and week 4 (n = 14). Raw data, shown as a triangle, surrounded by the box demonstrates the distribution of 25–75% of the data, with the whiskers representing the non-outlier data, and the horizontal line depicting the median. The dotted line connects the median points. Wilcoxon signed rank test was performed comparing subjects with week 0 and week 4 (n = 12).

We postulated that the Mtb specific CD8^+^ T cell response would be associated with other surrogates of bacterial burden such as AFB smear grade, delayed clearance of Mtb from sputum, or persistent culture positivity. However, baseline AFB smear grade was not associated with the magnitude of Mtb specific CD4^+^ or CD8^+^ T cell responses. Given the relative imprecision of the AFB smear grade, we sought to determine if quantitative mycobacterial cultures (CFU) were associated with Mtb specific CD4^+^ or CD8^+^ T cell responses. Of the 50 subjects with baseline and follow up microbiologic data, 35 (70%) had quantitative mycobacterial cultures performed at baseline and 14 (28%) again at week 4. Given the paucity of data at 4 weeks and lack of matching blood draw at that time, only the baseline data were analyzed. Using a negative binomial model, we found that baseline quantitative mycobacterial cultures of sputum were not correlated with the magnitude of the Mtb specific CD4^+^ or CD8^+^ T cell response (data not shown). We also postulated that subjects with delayed mycobacterial clearance or treatment failure would have persistently elevated Mtb specific CD8^+^ T cell responses. However, as shown in Table 2, over one-half (57%) were smear negative and three-quarters (73.5%) were culture negative at 8 weeks. Furthermore, subjects had a dramatic decline by several logs in quantitative cultures by week 4 (Figure 5; *p* = 0.0005). For the subset that required 9 months of therapy due to AFB positive sputum at 8 weeks (n = 21), follow up cultures at nine months remained negative (data not shown). As a result, we were unable to establish a direct correlation between microbiologic measures of bacterial burden and the Mtb specific T cell response.

## Discussion

We have demonstrated that the estimated Mtb specific CD8^+^ T cell response decreased by 58.4% at 24 weeks, with the majority of the decrease noted (38.7%) at 8 weeks in subjects receiving successful antituberculosis treatment. Surrogates of bacterial burden and response to therapy for Mtb infection have enormous potential in clinical practice to improve the management of those exposed to the Mtb, and those with TB disease. Among those who have been infected, but do not have TB disease, such markers might serve to reflect bacterial eradication, containment, growth or new exogenous infection. In this regard, such a marker might facilitate the identification of those who will progress to TB or, among subjects with TB disease, provide early identification of individuals at risk for treatment failure or relapse after treatment. Because CD8^+^ T cells recognize antigen derived from the intracellular environment, it is biologically plausible that they may discern a gradient of infection and distinguish infection from exposure. In this regard, the Mtb specific CD8 response in animal models correlates with bacterial load as demonstrated by the correlation with in vivo growth of Mtb [16] and computed tomography and patho-histologic findings [17]. In addition, in a murine model of Mtb treatment, a reduction in the frequency of Mtb specific CD8^+^ T cells, with a shift toward a central memory phenotype, has been demonstrated [18]. Additionally, Mtb specific CD8^+^ T cells preferentially recognize cells heavily infected with Mtb [15] and the presence of a robust Mtb specific CD8^+^ T cell response discerns young children with TB from their healthy, age matched controls with extensive environmental exposure [19]. Thus, we hypothesized that the CD8^+^ T cell response by virtue of sensing intracellular mycobacterial antigen may be a surrogate marker of response to therapy, and should decline with appropriate antituberculosis treatment.

To our knowledge, this is the first study in humans to prospectively evaluate changes in the Mtb specific CD8^+^ T cell response from the peripheral blood during TB treatment. We demonstrate a significant difference in the Mtb specific CD8^+^ T cell response during antituberculosis treatment, with a decline of 58% at 24 weeks. Interestingly, the majority of the overall decline, nearly 39%, occurred within 8 weeks and was relatively unaffected by malnutrition. In contrast, we found that the Mtb specific CD4^+^ T cell response exhibited substantial variability. In a parallel multivariate analysis, the Mtb specific CD4^+^ T cell response showed a marginal difference over 24 weeks (*p* = 0.07), with the major effect demonstrated in a pairwise comparison at 0 and 24 weeks (*p* = 0.08). This degree of variability and the marginal decrease is consistent with previously published studies evaluating commercial IGRAs, which are based on the CD4^+^ response, during antituberculosis treatment[9], [10], [12], [13].

Our finding that there is an early decline in the Mtb specific CD8^+^ response during the initial phase of successful TB treatment suggests that the Mtb specific CD8^+^ T cell response, potentially by detecting intracellular bacterial burden, may be a more accurate, and direct surrogate of response to therapy than the CD4^+^ response. Alternatively, it is possible that the Mtb specific CD4^+^ T cell response is more confounded by other host factors such as malnutrition, although our results do not support malnutrition as the sole explanation for the poor association between the Mtb specific CD4+ T cell response and duration of treatment over 24 weeks of antituberculosis therapy. The effects of malnutrition on immune dysregulation are well known [32] and lack of weight gain specifically in patients with TB has been associated with negative clinical outcomes such as relapse [33]. Specifically, protein calorie malnutrition is associated with lymphopenia as well as anergy to PPD in animal models [34]. Further, in subjects presenting with TB, diminished IFN-γ production in response to Mtb antigens has been observed, a defect that persisted for as long as a year following the initiation of TB treatment [31]. While the study by Hirsch *et al*. did not delineate CD4^+^ versus CD8^+^ T cell responses, the cohort was similar to the one presented herein in that patients presented with advanced disease, and were likely malnourished. Interestingly, we were able to follow the Mtb specific T cell responses of 11 malnourished subjects (BMI≤17) and show that some subjects were able to reconstitute their CD4^+^ T cell responses by the end of treatment. Conversely, for many of the subjects, the Mtb specific CD8^+^ T cell response declined, as seen with subjects with a normal BMI. Although we demonstrate a differential effect of malnutrition on the CD4^+^ T cell response, we suspect that severe protein calorie malnutrition affects both the CD4^+^ and CD8^+^ T cell response and we were underpowered to fully assess the effect of malnutrition on the CD8^+^ T cell response. Additionally, the use of BMI as a surrogate for malnutrition may be enhanced in future studies by different measures of nutritional status, especially in women [35].

In the mouse model, removal of antigen leads T cells to transition from cytokine producing effector T cells to those without short-term effector capacity [36]. Consequently, it has been postulated that the commercial IGRAs might be useful in monitoring TB treatment [4]. In this regard, many studies have shown modest declines in IFN-γ release using either non-commercial [6], [37] or the commercial IGRAs [7], [9], [10], [12], [13]. However, the quantitative variability of these responses both within and between individuals is striking. We propose that the variability seen across studies is associated with the use of peripheral blood mononuclear cells, which are dominated by CD4^+^ T cells. Thus, as CD4^+^ T cells recognize antigen sampled from either the intracellular or extracellular milieu, a specific CD4^+^ T cell response may be secondary to prior antigen-sensitization by Mtb rather than ongoing, intracellular infection. Our study demonstrating persistence of Mtb specific CD4^+^ T cell responses until week 24 of therapy is concordant with prior reports suggesting that Mtb antigens or the response to these antigens may persist for long periods following treatment [38]. Regardless, our finding that the Mtb specific CD4^+^ T cell response was impacted by malnutrition may account for performance differences in IGRAs when used in low resource/high incidence versus high resource/low incidence settings.

Our study has several unsettled points that need further study. Neither AFB smear grade, nor quantitative cultures were associated with the frequency of Mtb specific CD8^+^ T cell responses. From these data, as well as the observation of high frequency CD8^+^ T cell responses in those with LTBI [39] we would suggest that there is not a linear relationship between the magnitude of the Mtb specific CD8^+^ T cell response and traditional markers of bacterial burden such as smear positivity, chest radiographs and quantitative cultures. We postulate that each individual has a CD8 “set point” which reflects the complex interplay of antigenic exposure, in conjunction with host factors such as the HLA background. Nonetheless, our findings are concordant with the observation that removal of antigen results in decreasing T cell frequencies, and help explain the observed reduction in CD8^+^ T cell frequency as a result of antituberculosis therapy. Further, because our clinical cohort improved on appropriate antituberculosis treatment, we were unable to evaluate subjects failing therapy. As a result, it remains to be determined whether or not monitoring of the Mtb specific CD8^+^ T cell response will be of use for the prediction of treatment failure or relapse following treatment. For instance, people with unrecognized drug resistance would be expected to have delayed microbial clearance, which might result in a persistently elevated CD8^+^ T cell response. In this regard, our study enrolled two subjects with drug resistance, both of whom were changed to appropriate therapy early during their treatment course and demonstrated good clinical, and microbiologic response. As discussed, an analysis with and without the inclusion of these subjects was performed, and there was no change in our results. Thus, we believe that these subjects also had a decrease in bacterial burden, having been switched early to appropriate therapy. Thus, we were unable to determine if monitoring the Mtb specific CD8 response would identify subjects with drug resistance with the current cohort.

A further limitation was the use of ESAT-6 and CFP-10 in conjunction with IFN-γ as the immunological endpoint. In this regard, identification of novel Mtb antigens that are either disease [40] or T cell specific might improve the specificity and sensitivity of the immune assessment of bacterial burden (Lewinsohn unpublished data). Similarly, a more sophisticated measurement of T cell phenotype and function could provide additional insight into antigenic persistence, and hence provide additional clinical utility. This potential is illustrated by the recent findings that T cell production of IL-2 is associated with bacterial clearance [41], [42].

In summary, we have demonstrated that the estimated Mtb specific CD8^+^ T cell response decreased by 58.4% at 24 weeks, with the majority of the decrease noted (38.7%) at 8 weeks in subjects receiving successful antituberculosis treatment. Our data support the hypothesis that the frequency of Mtb specific CD8^+^ T cells, potentially as consequence of decreasing intracellular mycobacterial antigens, declines with antituberculosis therapy and may prove to be a surrogate marker of response to therapy. In contrast, the association of the Mtb specific CD4^+^ response with treatment was marginal, and affected by underlying malnutrition. At present, additional research is needed into the Mtb specific CD8^+^ T cell response during treatment at earlier time points (prior to 8 weeks), in subjects who fail treatment, and in subjects with HIV/TB coinfection. Such research would help to evaluate the practical application of the frequency of Mtb specific CD8^+^ T cells as a reliable and useful surrogate marker of response to therapy that may be used alone or in combination with other immune surrogates of antigen load [43], [44].
